# The effect of folic acid intake on congenital anomalies. A systematic review and meta-analysis

**DOI:** 10.3389/fped.2024.1386846

**Published:** 2024-07-19

**Authors:** Natnael Moges, Ermias Sisay Chanie, Rahel Mulatie Anteneh, Melkamu Aderajew Zemene, Asaye Alamneh Gebeyehu, Melaku Ashagrie Belete, Natnael Kebede, Denekew Tenaw Anley, Anteneh Mengist Dessie, Ermiyas Alemayehu, Fentaw Teshome Dagnaw, Zufan Alamrie Asmare, Sintayehu Simie Tsega

**Affiliations:** ^1^ Department of Pediatrics and Child Health Nursing, College of Health Sciences, Debre Tabor University, Debre Tabor, Ethiopia; ^2^ Department of Public Health, College of Health Science, Debre Tabor University, Debre Tabor, Ethiopia; ^3^ Department of Medical Laboratory Sciences, College of Medicine and Health Sciences, Wollo University, Dessie, Ethiopia; ^4^ Department of Health Promotion, School of Public Health College of Medicine Health Sciences, Wollo University, Dessie, Ethiopia; ^5^ Department of Ophthalmology, School of Medicine and Health Science, Debre Tabor University, Debre Tabor, Ethiopia; ^6^ Department of Medical Nursing, School of Nursing, College of Medicine and Health Science, University of Gondar, Gondar, Ethiopia

**Keywords:** effect, folic acid intake, congenital anomalies, systematic review and meta-analysis, folic acid

## Abstract

**Background:**

Congenital anomalies pose a significant challenge to global health and result in considerable morbidity and mortality in early childhood. With the decline of other causes of death among children under five, the burden of congenital anomalies is rising, emphasizing the need for improved prenatal care, screening, and nutrition for pregnant women. This systematic review and meta-analysis aim to estimate the pooled effect of folic acid intake on congenital anomalies.

**Methods:**

To identify relevant research published up until December 30/2023, we conducted electronic searches of PubMed/Medline, PubMed Central, Hinary, Google, African Journals Online, Web of Science, Science Direct, and Google Scholar databases using predefined eligibility criteria. We used Excel to extract data and evaluated the studies using the JBI appraisal checklist. We computed the pooled effect size with 95% confidence intervals for maternal folic acid intake on congenital anomalies using STATA version 17 and the DerSimonian and Laird random effects meta-analysis model. We assessed statistical heterogeneity using Cochran's *Q*-test, *I*^2^ statistic, and visual examination of the funnel plot.

**Results:**

The review included 16 case-control, cohort, and cross-sectional studies. According to the results of this systematic review and meta-analysis, maternal folic acid intake significantly lowers the incidence of congenital anomalies (odds ratio (OR), 0.23; confidence interval (CI), 0.16, 0.32). Among the included studies, both the Cochrane *Q*-test statistic (*χ*2 = 118.82, *p* < 0.001) and *I*^2^ test statistic (*I*^2^ = 87.38%, *p* < 0.001) revealed statistically significant heterogeneity. Egger's weighted regression (*p* < 0.001) and funnel plot show evidence of publication bias in this meta-analysis.

**Conclusion:**

The results of the recent meta-analysis and systematic review have demonstrated a significant association between maternal folic acid intake and the risk of congenital anomalies. Specifically, children whose mothers received periconceptional folic acid supplementation had a 77% reduced risk of congenital anomalies. To further investigate the correlation between maternal folic acid supplementation and the occurrence of various congenital anomalies, particularly in developing countries, it is recommended that a comprehensive prospective study be conducted.

**Systematic Review Registration:**

https://www.crd.york.ac.uk/prospero/, PROSPERO (CRD42024511508).

## Introduction

Congenital anomalies are a significant burden on global health, causing substantial morbidity and mortality in early life (1, 2). They are described as abnormalities of either structure or function that develop during intrauterine life and can be detected during pregnancy, at birth, or occasionally later in infancy. Congenital anomalies may appear as malformations, deformations, disruptions, sequences, dysplasias, or variants, each with distinct characteristics and root causes (3). Although the exact etiology of around half of congenital anomalies is unknown, known causes include chromosomal abnormalities, single gene errors, multifactorial inheritance, environmental teratogens, and micronutrient shortages (4). Even though the under-5 death rate is declining, congenital defects continue to be a major cause of neonatal and under-5 deaths (4). Congenital abnormalities have a substantial impact on mortality in Europe; they are responsible for up to 49% of deaths in children aged 1%–9% and 71% of deaths in neonates (5). Furthermore, Congenital abnormalities are a major burden in low-income nations; they account for 2.8% of neonatal admissions and 8.6 per 1,000 births, with a 33.2% neonatal death rate (6).

Congenital anomalies cause long-lasting disability and health problems. These conditions can lead to extended hospital stays, recurrent infections, neurological and psychological issues, and the need for significant surgical intervention. As other causes of death among children under five declines, the burden of congenital anomalies is increasing. This underscores the importance of improved prenatal care, screening, and nutrition for pregnant women (4, 7).

The US Public Health Service advised daily folic acid supplementation with 0.4 mg for all women who potentially become pregnant when persistent evidence of the protective benefit of folic acid supplementation against NTDs appeared (8). International recommendations advise women to take 0.4 mg of folic acid supplements from the time they are trying to conceive (at least 4 weeks) until 12 weeks into their pregnancy (4). Many women still do not take the recommended folic acid supplements during pregnancy, especially those from lower socioeconomic backgrounds, despite legislation in various nations regarding this matter (9, 10).

The need for micronutrients increases substantially during pregnancy, but they become particularly crucial in the first trimester when organogenesis is most active (11). The growth and development of the fetus are impacted by the nutritional status both before and after conception, and deficits raise the chance of birth abnormalities (12). Along with iron (13), iodine (14), and vitamin D (15), folate deficiency is one of the most prevalent micronutrient deficits among women in reproductive age (11).

Both experimental and observational studies have shown the efficacy of folic acid supplementation throughout periconception and pregnancy in lowering the risk of neural-tube abnormalities in offsprings (16–18). Besides, numerous studies have investigated the potential links between folic acid and multivitamins and other birth defects, including urinary tract defects, congenital heart defects, limb reduction defects, and other structural developmental anomalies (19–21). However, based on our current understanding, the overall impact of folic acid intake on congenital anomalies has not been thoroughly explored in prior research. Hence, the aim of this systematic review and meta-analysis is to assess the pooled influence of folic acid consumption on congenital abnormalities.

## Methods

### Reporting of the findings and review registration

The current systematic review and meta-analysis were reported using Preferred Reporting Items for Systematic Reviews and Meta-Analyses guidelines (22) (Supplementary File S1). Using the registration ID CRD42024511508, the review protocol was registered in PROSPERO.

### Searching techniques

We conducted a thorough search across numerous databases, including African Journals Online, Web of Science, Google Scholar, Hinary, PubMed/MEDLINE, Science Direct and PubMed Central, until December 30, 2023. We even perused the articles' reference lists that we could find. Using phrases from the Medical Subject Heading, we conducted the primary search on PubMed. We searched all of the databases for the same information, then we utilized Google and Google Scholar to get any additional information (Supplementary File S2).

### Inclusion and exclusion criteria

This analysis includes all observational studies (cross-sectional, cohort and case-control) that described how congenital anomalies were impacted by folic acid intake. This meta-analysis employed the Cruds odd ratios and included studies that reported the connection using odds ratios. Exclusions from the current study included systematic reviews and meta-analyses, non-human studies, studies not disclosing the outcome of interest, conference proceedings, qualitative studies, case reports, editorial comments, and studies done in languages other than English. Furthermore, studies for which we could not identify the original data were removed since they did not offer odds ratios based on two-by-two tables.

### Data extraction

All required data was separately extracted by two authors (NM and ESC) using a Microsoft Excel data extraction template. First author names, publication year, research sites, study time, study designs, sample size, case classification data, exposure and outcome information, and adjusted ORs/RRs with matching CIs were among the significant data that were extracted. We derived a crude estimate in cases where adjusted estimates were not available. In the event that a study did not provide an estimate of relative risk (OR), we used conventional equations to calculate ORs, RRs, and 95% confidence intervals from the raw data that was reported in the study. Through dialogue, disagreements that arose during the data extraction process were settled, and the two authors came to a mutual understanding.

### Quality evaluation

The quality of each study was evaluated using the JBI quality rating checklist (23). For cross-sectional, case control and cohort studies that reported the association of folic acid intake and congenital anomalies, an adaptation of the eight, ten and eleven-item JBI critical evaluation checklist was made respectively (Supplementary File S3). Two reviewers (NM, ESC) assessed each study's quality independently using the framework. Disagreements between reviewers were settled during the quality assessment process by averaging the two reviewers' scores. Ultimately, a study was classified as low risk if it scored five or higher on every quality evaluation criterion (24).

### Statistical analysis

For additional analysis, extracted data from Microsoft Excel spreadsheets were imported into STATA/SE for Windows version 17. Using the DerSimonian (25) and Laird random effects meta-analysis (random effects model), the pooled odds ratio with 95% CI of maternal folic acid uses on congenital anomalies was computed. The subgroup and sensitivity analyses were employed to confirm the potential causes of the heterogeneities among the studies that were included. Alongside the pooled estimates were their 95% confidence intervals. Forest plots, summary tables, and text were used to display the meta-analysis results.

### Publication bias and heterogeneity

By examining the asymmetries, publication bias was examined and confirmed at a 5% significant level using Egger's regression test (26). The Cochrane Q statistics, the *I*^2^ test, and the forest plot were employed to identify study heterogeneity (27). The *I*^2^ values of 25%, 50%, and 75%, respectively, were considered to have low, medium, and high heterogeneity (28). Heterogeneity in this study was deemed significant when the *p*-value was less than 0.05 and the *I*^2^ value was greater than 50%. Additionally, sensitivity and sub-group analyses were used to address potential sources of significant heterogeneity.

## Results

### Retrieved research

**Figure 1:**
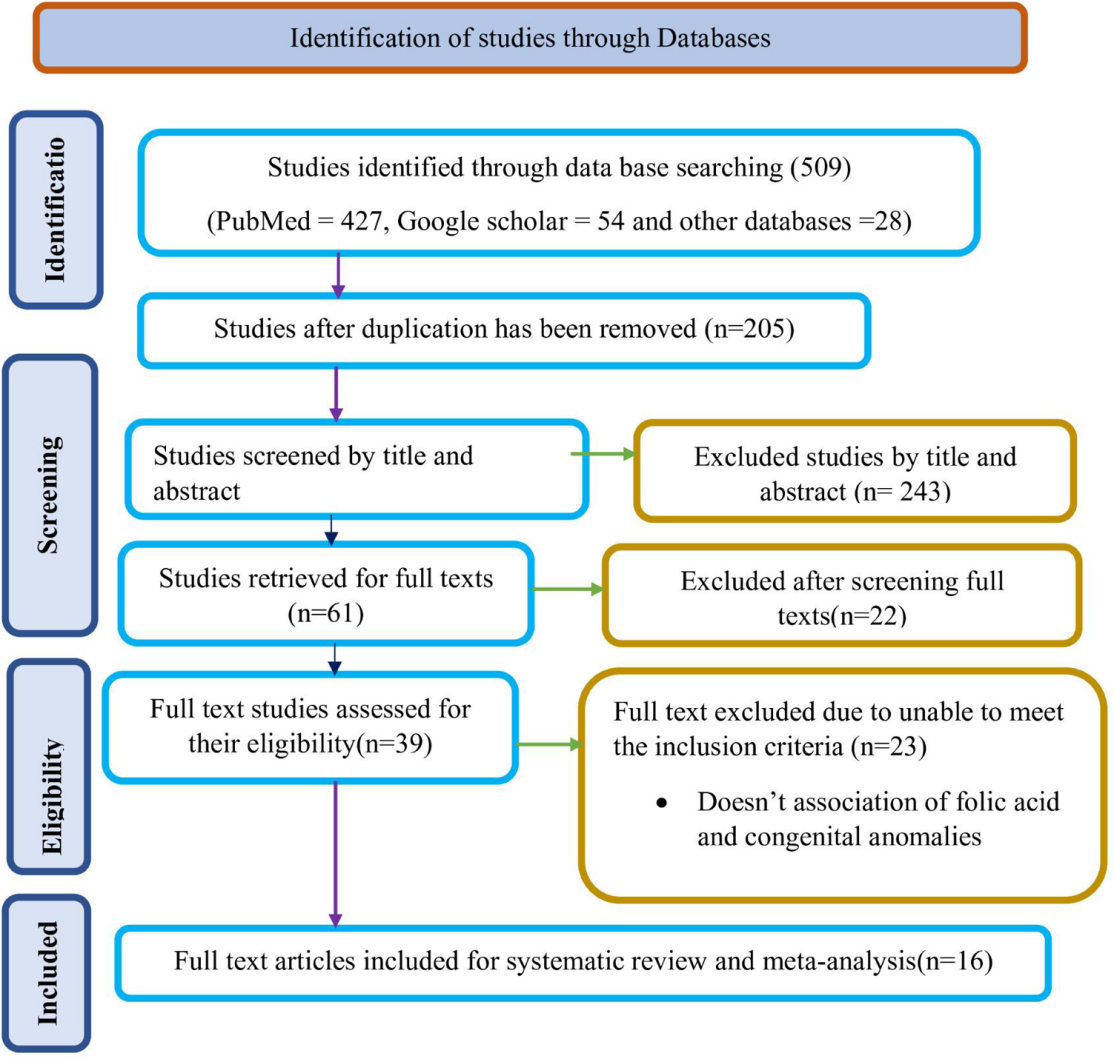
Study selection flow diagram; a figure adapted from the PRISMA group statement for this review. PRISMA, preferred reporting items for systematic reviews and meta analyses.

After conducting a preliminary search using specified databases, 509 study findings were discovered. Following the deletion of redundant results, 304 reports remained. Subsequently, 243 of these reports were excluded based on a preliminary screening of their titles and/or abstracts, as they were deemed irrelevant. This was because the majority of the papers' titles and/or abstracts were unrelated to the current issue, and the titles and/or abstracts of the remaining studies discussed the impact of folic acid supplementation on other specific birth defects. When applying established inclusion and exclusion criteria, the researchers evaluated the full text of 61 publications, removing 22 studies that were deemed ineligible. After examining the remaining 23 articles in their entirety, 16 studies were found to be relevant to the review (Figure 1).

### Description of included studies

The included articles in this systematic review and meta-analysis were published between 2011 and 2023. Nearly half of the 16 included research were carried out in Ethiopia. Specifically, 9 studies were found in Ethiopia (29–37) and two studies from Tanzania (38, 39).The remaining studies are obtained from one in China (40), one in Lebanon (41),one in Egypt (42),one in Nigeria (43), and one in Saudi Arabia (44). Regarding the study designs of the included articles 8(47%) are cross sectional (30, 31, 35, 36, 39, 41, 43, 45). The remaining 6 studies are case control (29, 32–34, 37, 38) and three cohort studies (40, 42, 44). The sample size of the included studies is ranged from 219 with the use of cross-sectional study and 660,280 with the use of cohort studies (Table 1).

**Table 1:** A summary of the features of articles that were part of this systematic review and meta-analysis.

Study	Year	Country	Study design	Study period	Sample size	Confounders adjusted	Quality
Dong et al. (40)	2023	China	Cohort	2017–2020	17,713	Yes	Low risk
Shawky et al. (42)	2011	Egypt	Cohort	1995–2009	660,280	Yes	Low risk
Belama et al. (29)	__	Ethiopia	Case –control	2022	387	Yes	Low risk
Birhanu et al. (31)	2021	Ethiopia	Cross sectional	2020	422	Yes	Low risk
Gedamu et al. (30)	2021	Ethiopia	Cross sectional	2018–2019	2,218	Yes	Low risk
Getachew et al. (36)	2022	Ethiopia	Cross sectional	2018	754	Yes	Low risk
Jemal et al. (34)	2021	Ethiopia	Case –control	2020	418	Yes	Low risk
Abebe et al. (37)	2021	Ethiopia	Case –control	2016–2018	251	Yes	Low risk
Tsehay et al. (32)	2019	Ethiopia	Case –control	2017–2018	398	Yes	Low risk
Taye et al. (33)	2018	Ethiopia	Case –control	2015	414	Yes	Low risk
Adane et al. (35)	2018	Ethiopia	Cross sectional	2017–2018	19,650	Yes	Low risk
Francine et al. (41)	2014	Lebanon	Cross sectional	2009	1,000	Yes	Low risk
Ajao et al. (43)	2019	Nigeria	Cross sectional	2012–2016	1,057	Yes	Low risk
Kurdi et al. (44)	2019	Saudi Arabia	Cohort	2010–2013	28,646	Yes	Low risk
Mashuda et al. (39)	2014	Tanzania	Cross sectional	2012–2013	445	Yes	Low risk
Kishimba (38)	2015	Tanzania	Case -control	2011–2012	400	Yes	Low risk

### Quality evaluation

The JBI quality appraisal standards were utilized to assess the quality of all the studies that were included. A total of 16 papers were assessed using the evaluation checklist for cross-sectional, case-control, and cohort studies. This checklist comprises eight, ten, and eleven questions respectively, and the responses to these questions are categorized as yes, no, uncertain, or not applicable. The quality evaluation grade for each study was determined by employing the JBI descriptors for each item. Consequently, the quality scores of the studies ranged from seven to ten. Thus, it is highly unlikely that any of the studies would be of poor quality (29–44) (Supplementary File S4).

### Effect of folic acid intake on congenital anomalies

**Figure 2:**
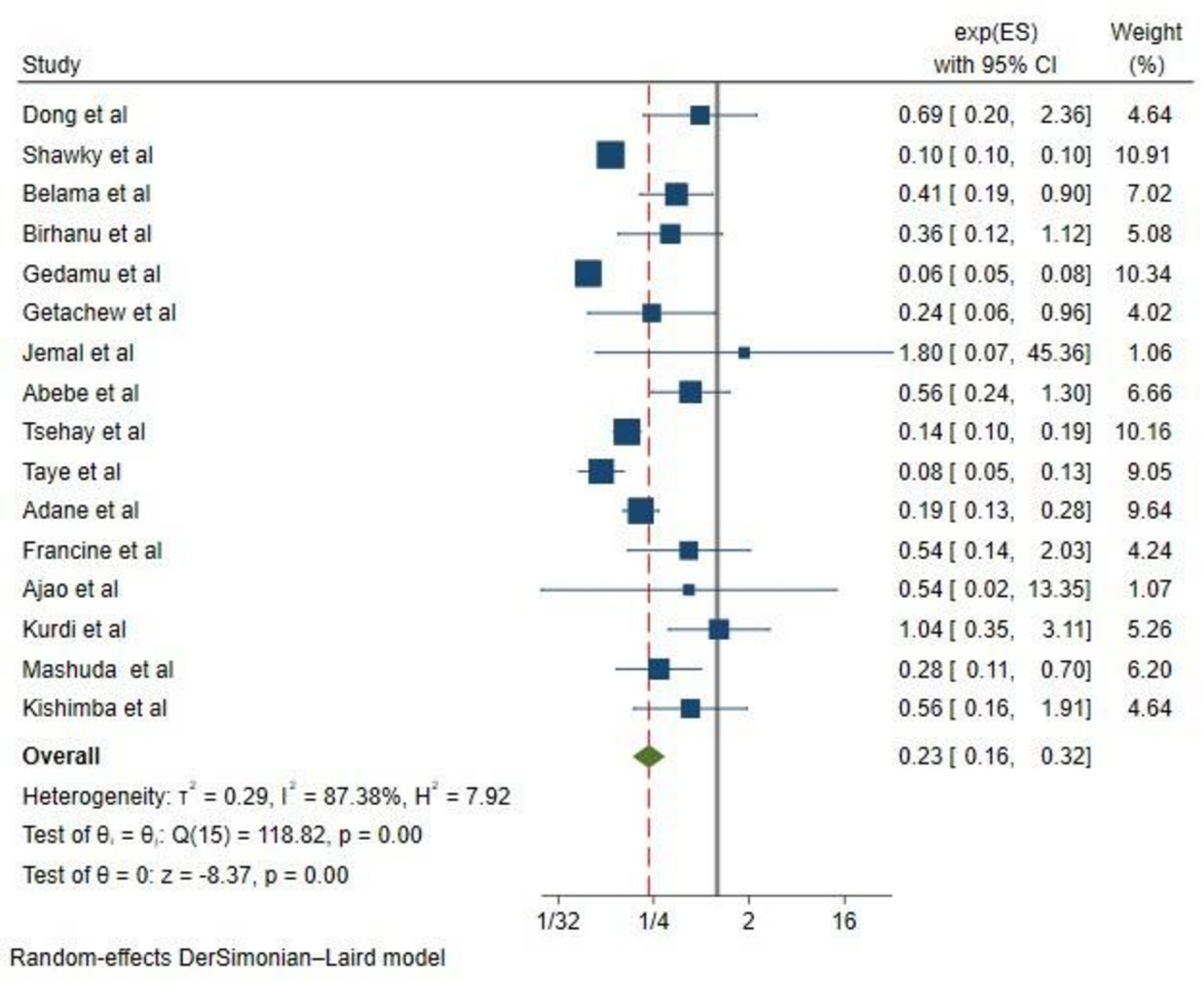
The forest plot of the 16 studies that are included shows the link between folic acid intake and congenital anomalies. The bars show the appropriate 95% CIs, and the size of the square is proportionate to the accuracy of the study-specific effect estimates. The width of the diamond represents the relevant 95% CI, and the diamond is centered on the summary effect size of all included studies.

In the random effects model, the pooled relative risk of congenital anomalies for children born to mothers who took folic acid was 0.23 (0.16, 0.32) higher than for children born to mothers who did not take folic acid. Overall, the results of this systematic review and meta-analysis show that taking supplements of folic acid prior to conception (at least 4 weeks) and during pregnancy until 12 weeks significantly reduces the risk of congenital anomalies by 77% (OR, 0.23; CI, 0.16–0.32) (Figure 2).

### Heterogeneity and publication bias

**Figure 3:**
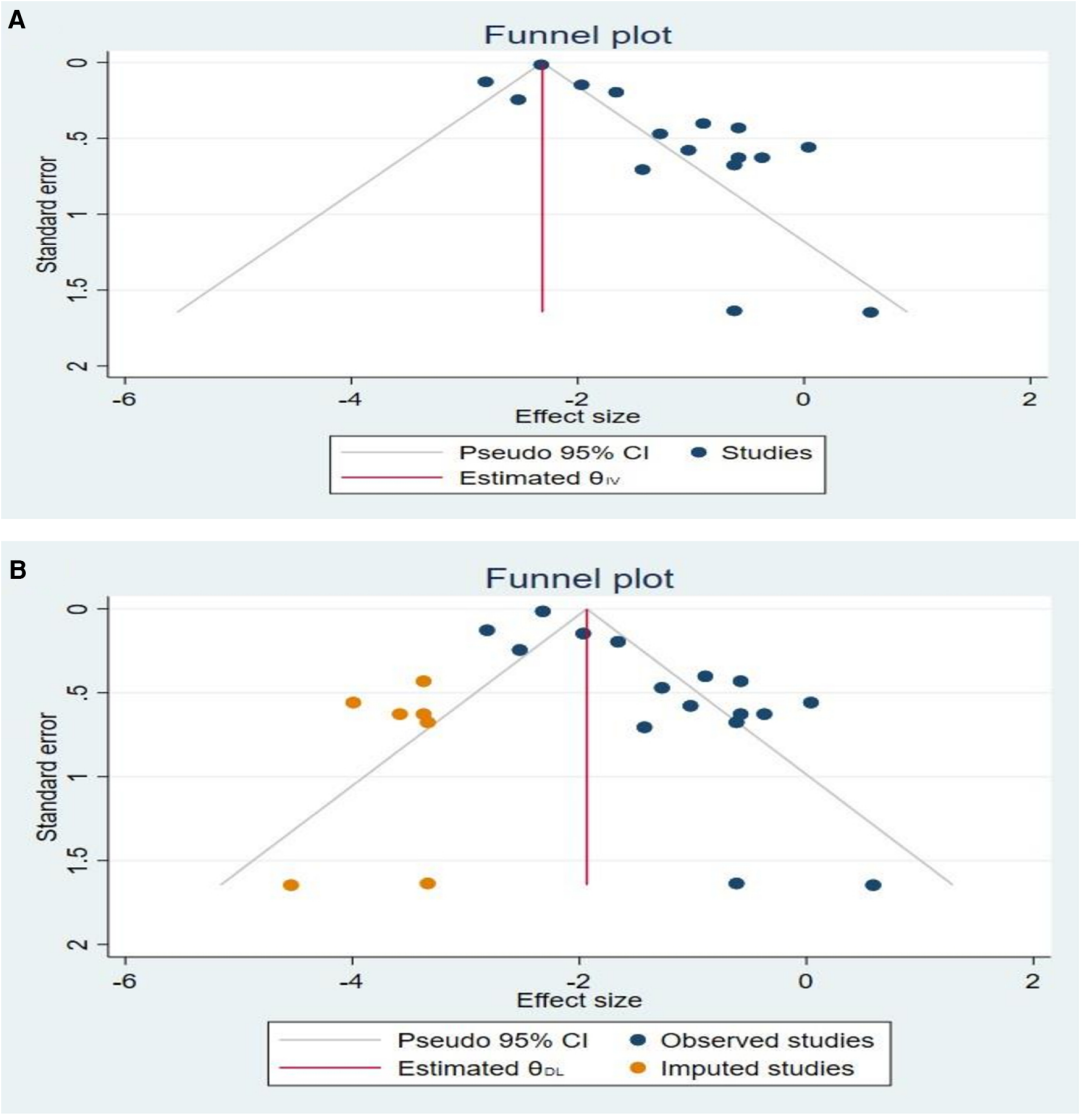
(**A**) A funnel plot with a pseudo 95% confidence limit used to test for publication bias. (**B**) A funnel plot with a pseudo 95% confidence limit after a trim-and-fill analysis in which seven studies have been imputed.

Because of the significant heterogeneity among the included studies (*I*^2^ = 87.38%, *p* < 0.001), the pooled odds ratio was calculated using the random effect model. Egger's regression test and the funnel plot were used to investigate the potential causes of the increased heterogeneity. The funnel plot was determined to be asymmetrical, and the Egger's regression test objectively confirmed this by showing that the bias *p*-value was *p* < 0.001 and the funnel plot was statistically significant (Figure 3A).

A trim and fill study was then conducted. After adding the seven studies, the trim and fill analysis yielded a pooled prevalence of 0.14 (95% CI: 0.11–0.20) and it was discovered that seven imputed studies may be the source. We had filled in two trials using the run L0 estimate. Besides, a funnel plot based on trim fill analysis was created (Figure 3B).

**Figure 4:**
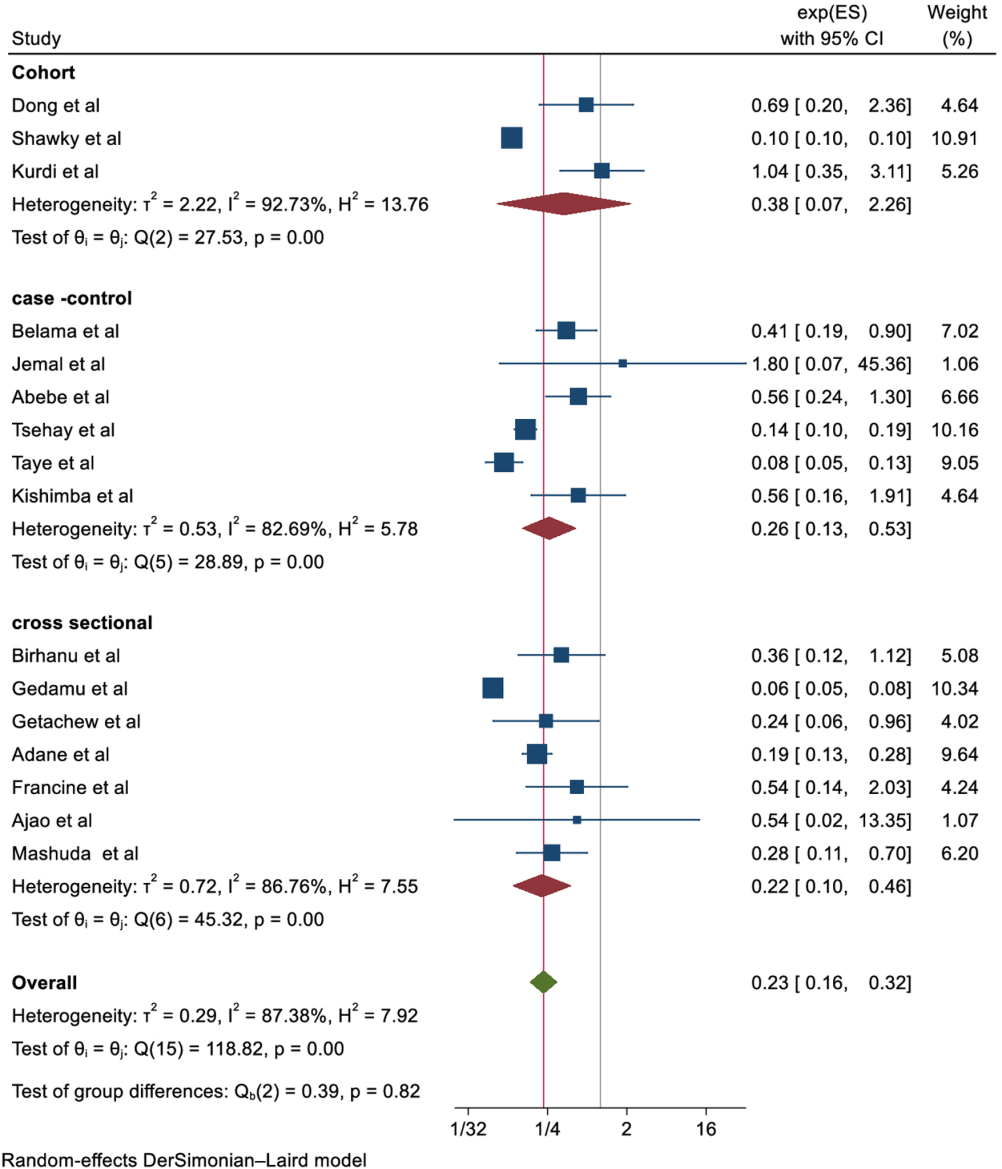
Subgroup analysis based on study design. ES, Effect size.

**Figure 5:**
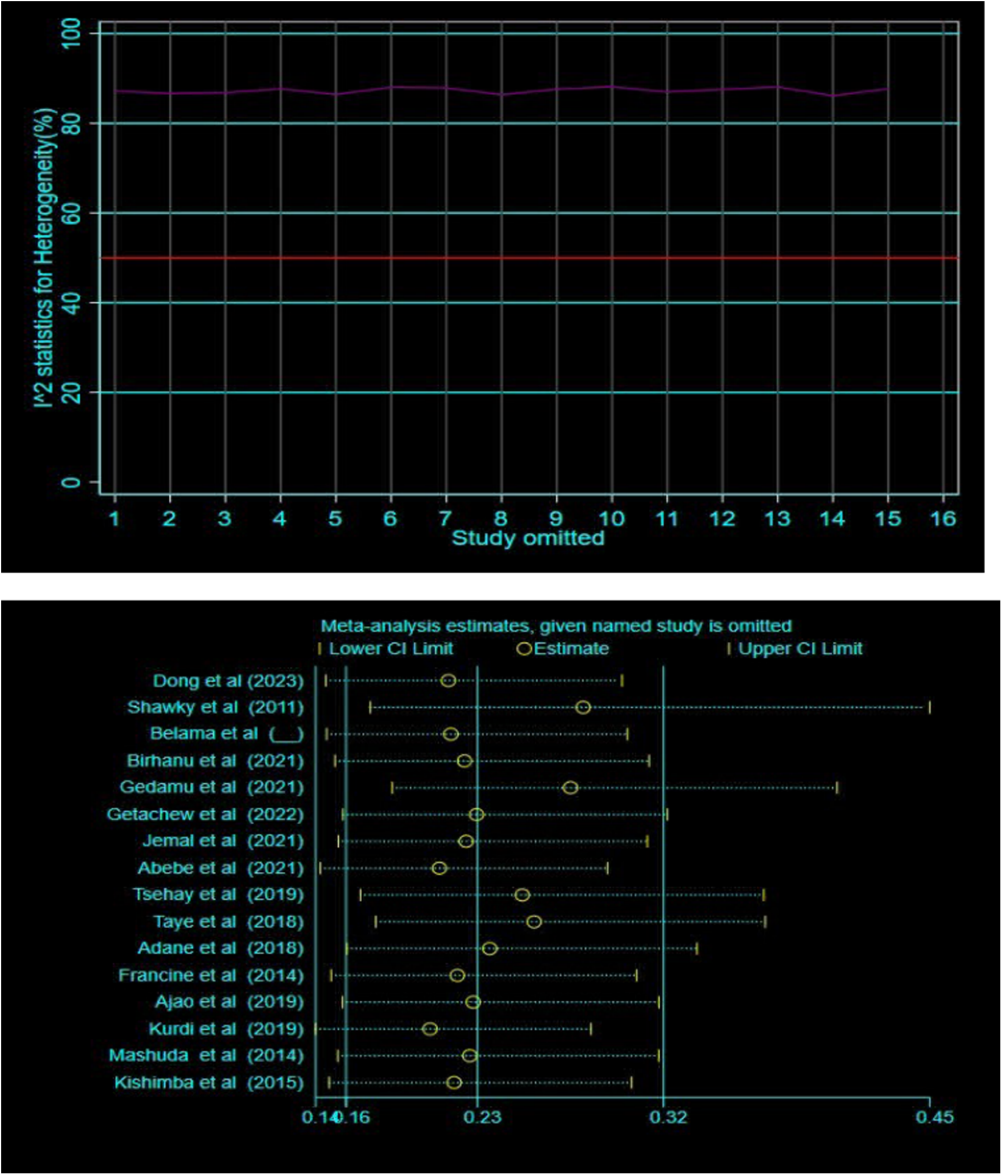
Sensitivity analysis of the effect of folic acid intake and congenital anomalies using metaplot and metaninf STATA command respectively.

Additionally, subgroup and sensitivity analyses were conducted to investigate the potential sources of heterogeneity. Nevertheless, heterogeneity persisted high within the subgroup estimates (Figure 4). The sensitivity analysis also suggested that no single study was responsible for this significant heterogeneity (Figure 5).

## Discussion

By integrating investigations conducted between 2011 and March 2023, this comprehensive meta-analysis, to the best of our understanding, represents an exhaustive scholarly endeavor that delineated the cumulative impact of maternal supplementation of folic acid on the occurrence of congenital anomalies. It was determined that folic acid supplementation substantially diminished the prevalence of congenital anomalies in the offspring, as evidenced by the overall and majority of the subgroup analysis findings of the present systematic review and meta-analysis (OR, 0.23; CI, 0.16–0.32). The outcomes of this systematic review and meta-analysis revealed that the vulnerability to various forms of birth defects might be reduced by 77% through the administration of folic acid supplements immediately prior to and during the initial trimester of pregnancy.

Vitamin B9, or folic acid, is a water-soluble vitamin that is essential to the body. It is crucial during times of fast growth, such as infancy, adolescence, and pregnancy, as it plays a role in the synthesis of DNA and RNA, the body's genetic material (46). Folate deficiency plays in atherosclerotic cardiovascular disease, neurological and neuropsychiatric disorders, congenital defects, and carcinogenesis. Folate has been identified as having great potential to prevent a wide range of disorders through folate supplementation (47).

Through a number of different processes, folate deprivation during pregnancy can raise the chance of congenital abnormalities. First and foremost, folate is essential for the synthesis and repair of DNA, as well as for healthy cell division and tissue development throughout the embryonic stage (48). A folate deficit can cause disruptions in DNA synthesis and repair systems, which can increase susceptibility to genetic mutations and cause genomic instability. This can have an impact on the development of different organs and tissues. Furthermore, through its effects on histone modifications, microRNA expression, and DNA methylation patterns, folate plays a critical role in epigenetic control (49). Gene expression patterns related to organogenesis, tissue differentiation, and embryonic development can be dysregulated by a folate deficit. The development of congenital anomalies like heart problems, limb abnormalities, and facial dysmorphisms can be facilitated by these changes in gene expression profiles, which can interfere with normal developmental processes (50, 51).

Moreover, oxidative stress and inflammation brought on by a folate shortage are linked to the pathophysiology of congenital abnormalities (47). Because folate plays a crucial role in the metabolism of homocysteine, a lack of it can raise homocysteine levels, which can cause oxidative damage to tissues and cells. Through apoptosis induction, tissue morphogenesis impairment, and interference with cellular signaling pathways, oxidative stress and inflammation can cause developmental disruptions in embryos. Furthermore, a lack of folate has a negative effect on neurodevelopment, which is necessary for healthy neuronal migration, proliferation, and differentiation. A lack of folate can cause abnormalities in the development of the brain, which can lead to congenital malformations such hydrocephalus, microcephaly, and intellectual impairments (46). These complex pathways demonstrate the extensive effects of folate deprivation on the development of the embryo and the elevated risk of congenital defects.

As previously discussed, there is a higher chance of birth abnormalities in children born to pregnant mothers who do not get enough folic acid. Doses of folic acid supplements for pregnant mothers usually vary based on personal health status, food consumption, and particular physician advice. Nonetheless, the standard recommendation for all pregnant mothers is to take a 400–800 microgram (mcg) prenatal vitamin every day, ideally beginning at least one month before to conception and continuing through the first trimester of pregnancy (52). Besides, Centers for Disease Control and Prevention (CDC) advises all women of reproductive age to take 400 mcg of folic acid daily from dietary supplements or fortified meals. The CDC recommends that pregnant women take 600 micrograms of folic acid each day in the form of supplements in addition to eating foods high in folate.

This systematic review and meta-analysis possess inherent limitations, akin to any other study. Consequently, it is imperative to consider these limitations when analyzing the data. The initial constraint of this review was its restriction to English articles or reports, which means that findings from publications in different languages could potentially influence our own findings.

Moreover, the expected report may be impacted by the diversity in study designs, sample sizes, study locations, and publication years. Thus, the interpretation of these study findings should take into account this variability.

Furthermore, the assessment encompassed investigations from a limited number of nations due to the scarcity of available data on the correlation between congenital malformations and congenital anomalies. The study's strength was the greatest effort made to locate papers from various databases and grey literatures, as well as the thorough analysis carried out to compute pooled estimates.

Inconclusion, according to the current systematic review and meta-analysis, there is a significant correlation between maternal folic acid supplementation and the risk of congenital anomalies. The risk is lowered by 77% for those who take folic acid. Furthermore, research indicates that a substantial proportion of reproductive-age women, especially those in underdeveloped nations, may not use or consume foods enriched with folic acid. Therefore, we advise the implementation of a large-scale cohort study to examine the impact of maternal periconceptional folic acid supplementation on the incidence of different forms of congenital anomalies in mothers residing in low-income communities or nations.

## Data Availability

The original contributions presented in the study are included in the article/Supplementary Material, further inquiries can be directed to the corresponding author.
