# Supplementary material for: The effect of folic acid intake on congenital anomalies. A systematic review and meta-analysis
Source: Front Pediatr. 2024 Jul 19;12:1386846. doi: 10.3389/fped.2024.1386846 (PMC11294162; doi:10.3389/fped.2024.1386846)
Supplement: Supplementary file 1 [file Datasheet1.pdf]

JBI Critical Appraisal Checklist

JBI Critical Appraisal Checklist for Studies Reporting Prevalence Data

Reviewer ______________________ Date ____________________

Author ________________________ Year ___________ Record Number ______

	Yes	No	Unclear	Not applicable
1. Was the sample frame appropriate to address the target population?	☐	☐	☐	☐
2. Were study participants sampled in an appropriate way?	☐	☐	☐	☐
3. Was the sample size adequate?	☐	☐	☐	☐
4. Were the study subjects and the setting described in detail?	☐	☐	☐	☐
5. Was the data analysis conducted with sufficient coverage of the identified sample?	☐	☐	☐	☐
6. Were valid methods used for the identification of the condition?	☐	☐	☐	☐
7. Was the condition measured in a standard, reliable way	☐	☐	☐	☐

for all participants?

8. Was there appropriate statistical analysis? ☐ ☐ ☐ ☐

9. Was the response rate adequate, and if not, was the low ☐ ☐ ☐ ☐

response rate managed appropriately?

Overall appraisal: Include ☐ **Exclude** ☐ **Seek further info** ☐

Comments (Including reason for exclusion)

____

____

____

JBI Critical Appraisal Checklist for Analytical Cross Sectional Studies

Reviewer ______________________ Date ____________________

Author ________________________ Year ___________ Record Number ______

	Yes	No	Unclear	Not applicable
1. Were the criteria for inclusion in the sample clearly defined?	☐	☐	☐	☐
2. Were the study subjects and the setting described in detail?	☐	☐	☐	☐
3. Was the exposure measured in a valid and reliable way?	☐	☐	☐	☐
4. Were objective, standard criteria used for measurement of the condition?	☐	☐	☐	☐
5. Were confounding factors identified?	☐	☐	☐	☐
6. Were strategies to deal with confounding factors stated?	☐	☐	☐	☐
7. Were the outcomes measured in a valid and reliable way?	☐	☐	☐	☐

8. Was appropriate statistical analysis used? ☐ ☐ ☐ ☐

Overall appraisal: Include ☐ Exclude ☐ Seek further info ☐

Comments (Including reason for exclusion)

____

____

____

JBI Critical Appraisal Checklist for Case Control Studies

Reviewer ________________________ Date ______________________

Author __________________________ Year ____________ Record Number _______

		Yes	No	Unclear	Not applicable
1.	Were the groups comparable other than the presence of disease in cases or the absence of disease in controls?	☐	☐	☐	☐
2.	Were cases and controls matched appropriately?	☐	☐	☐	☐
3.	Were the same criteria used for identification of cases and controls?	☐	☐	☐	☐
4.	Was exposure measured in a standard, valid and reliable way?	☐	☐	☐	☐
5.	Was exposure measured in the same way for cases and controls?	☐	☐	☐	☐

of the identified sample?

6. Were confounding factors identified? ☐ ☐ ☐ ☐

7. Were strategies to deal with confounding factors stated? ☐ ☐ ☐ ☐

8. Were outcomes assessed in a standard, valid and reliable way for cases and controls? ☐ ☐ ☐ ☐

9. Was the exposure period of interest long enough to be meaningful? ☐ ☐ ☐ ☐

10. Was appropriate statistical analysis used? ☐ ☐ ☐ ☐

Overall appraisal: Include ☐ **Exclude** ☐ **Seek further info** ☐

Comments (Including reason for exclusion)

____

____

____

JBI Critical Appraisal Checklist for Cohort Studies

Reviewer ______________________ Date ____________________

Author ________________________ Year ___________ Record Number ______

	Yes	No	Unclear	Not applicable
1. Were the two groups similar and recruited from the same population?	☐	☐	☐	☐
2. Were the exposures measured similarly to assign people to both exposed and unexposed groups?	☐	☐	☐	☐
3. Was the exposure measured in a valid and reliable way?	☐	☐	☐	☐
4. Were confounding factors identified?	☐	☐	☐	☐
5. Were strategies to deal with confounding factors stated?	☐	☐	☐	☐
6. Were the groups/participants free of the outcome at the start of the study (or at the moment of exposure)?	☐	☐	☐	☐
7. Were the outcomes measured in a valid and reliable way?	☐	☐	☐	☐

8. Was the follow up time reported and sufficient to be long enough for outcomes to occur? ☐ ☐ ☐ ☐

9. Was follow up complete, and if not, were the reasons to loss to follow up described and explored? ☐ ☐ ☐ ☐

10. Were strategies to address incomplete follow up utilized? ☐ ☐ ☐ ☐

11. Was appropriate statistical analysis used? ☐ ☐ ☐ ☐

Overall appraisal: Include ☐ **Exclude** ☐ **Seek further info** ☐

Comments (Including reason for exclusion)

____

____

____
