# Supplementary material for: The effect of folic acid intake on congenital anomalies. A systematic review and meta-analysis
Source: Front Pediatr. 2024 Jul 19;12:1386846. doi: 10.3389/fped.2024.1386846 (PMC11294162; doi:10.3389/fped.2024.1386846)
Supplement: Supplementary file 3 [file Datasheet3.pdf]

PubMed Searching Methods

S.no.	Searching terms	Number of articles
**#1.**	“Association” [Mesh] OR associate*[tw] OR connection*[tw]	2,366,362
**#2.**	"Folic Acid"[Mesh] OR “folic acid intake” [tw] OR “folic acid supplementation*” [tw] OR Multivitamin*[tw]	**1**3843
**#3.**	"Congenital Abnormalities"[Mesh] OR “birth defect*” [tw] OR “congenital anomalie*” [tw]	**1**36580
	**#1 AND #2 AND #3**	**4**27
