# Supplementary material for: The effect of folic acid intake on congenital anomalies. A systematic review and meta-analysis
Source: Front Pediatr. 2024 Jul 19;12:1386846. doi: 10.3389/fped.2024.1386846 (PMC11294162; doi:10.3389/fped.2024.1386846)
Supplement: Supplementary file 4 [file Datasheet4.pdf]

Supplementary file 4: (A) The quality status of studies based on JBI critical appraisal checklist for Cohort studies

Studies	Were the two groups similar and recruited from the same population?	Were the exposures measured similarly to assign people to both exposed and unexposed group?	Was the exposure measured in a valid and reliable way?	Were confounding factors identified??	Were strategies to deal with confounding factors stated ?	Were the groups/participants free of the outcome at the start of the study (or at the moment of exposure)?	Were the outcomes measured in a valid and reliable way?	Was the follow up time reported and sufficient to be long enough for outcomes to occur?	Was follow up complete, and if not, were the reasons to loss to follow up described and explored?	Were strategies to address incomplete follow up utilized?	Was appropriate statistical analysis used?	Total score out of 11
Dong et al	Yes	Yes	yes	yes	Yes	Yes	Yes	Yes	Yes	UC	Yes	10
Shawky et al	Yes	Yes	Yes	Yes	Yes	UC	Yes	Yes	yes	UC	Yes	9
Kurdi et al	Yes	Yes	Yes	Yes	Yes	UC	Yes	Yes	Yes	UC	Yes	9

Supplementary file 4: (B) The quality status of studies based on JBI critical appraisal checklist for cross-sectional studies

Studies	Were the criteria for inclusion in the sample clearly defined?	Were the study subjects and the setting described in detail?	Was the exposure measured in a valid and reliable way?	Were objective, standard criteria used for measurement of the condition?	Were confounding factors identified?	Were strategies to deal with confounding factors stated?	Were the outcomes measured in a valid and reliable way?	Was appropriate statistical analysis used?	Total, out of 8
Birhanu et al	Yes	Yes	yes	yes	Yes	Yes	UC	Yes	7
Gedamu et al	Yes	Yes	Yes	Yes	Yes	UC	UC	Yes	6
Getachew et al	Yes	Yes	Yes	Yes	Yes	UC	UC	Yes	6
Adane et al	Yes	Yes	Yes	Yes	Yes	Yes	UC	Yes	7
Francine et al	Yes	Yes	Yes	Yes	Yes	Yes	Yes	Yes	8
Ajao et al	Yes	Yes	Yes	Yes	Yes	Yes	UC	Yes	7
Mashuda et al	Yes	Yes	Yes	Yes	Yes	Yes	Uc	Yes	8

Supplementary file 4: (C) The quality status of studies based on JBI critical appraisal checklist for case control studies.

Studies	Were the group comparable?	Were cases and controls matched?	Were the same criteria for identification	Was exposure measured in a standard, valid and reliable way?	Was exposure measured in the same way ?	Were confounding factors identified?	Were strategies to deal the confounders stated?	Were outcomes assessed in a standard, valid and reliable way?	Was the exposure period of interest long enough	Was appropriate statistical analysis used?	Total out of 10
Belama et al	Yes	No	Yes	UC	Yes	Yes	Yes	Yes	UC	Yes	7
Jemal et al	Yes	No	Yes	UC	Yes	Yes	Yes	Yes	UC	Yes	7
Tsehay et al	Yes	NO	Yes	Yes	Yes	Yes	No	Yes	Yes	Yes	8
Abebe et al	Yes	No	Yes	Yes	Yes	Yes	Yes	Yes	UC	Yes	8
Kishimba et al	Yes	No	Yes	Yes	Yes	Yes	Yes	Yes	Yes	Yes	9
Taye et al	yes	no	Yes	Yes	Yes	Yes	Yes	Yes	UC	Yes	8
